# Acquired cystic disease-associated renal cell carcinoma with PTCH1 mutation: a case report

**DOI:** 10.3389/fonc.2024.1349610

**Published:** 2024-02-02

**Authors:** Luting Zhou, Haimin Xu, Yang Liu, Xiangyun Li, Chuanying Li, Xiaoqun Yang, Chaofu Wang

**Affiliations:** Department of Pathology, Ruijin Hospital, Shanghai Jiaotong University School of Medicine, Shanghai, China

**Keywords:** acquired cystic disease-associated renal cell carcinoma, clear cell renal cell carcinoma, next-generation sequencing, Ptch1, VHL

## Abstract

Acquired cystic disease-associated renal cell carcinoma (ACD-RCC) is an extremely rare kidney tumor seen mainly in patients with end-stage renal disease. Currently, there are few reports on this type of tumor. We describe the case of a 58-year-old man who had been receiving peritoneal dialysis for more than nine years due to chronic renal insufficiency and uremia. One year after undergoing left renal clear cell renal cell carcinoma resection, a space-occupying lesion was found in the right kidney for which he underwent right nephrectomy. The histopathology of this tumor showed solid or tubular cell arrangements, with some areas of cyst formation. Vacuoles of varying sizes were present in the cytoplasm, and varying amounts of calcium oxalate crystals were found in the tumor cells or interstitium. The pathological diagnosis was ACD-RCC. Next-generation sequencing detected mutations in the PTCH1, MTOR, FAT1, SOS1, RECQL4, and CDC73 genes in the right renal tumor. This is a rare case of a patient with ACD-RCC in the right kidney and clear cell renal cell carcinoma in the left kidney. The findings suggest that mutations in PTCH1 associated with ACD-RCC may have acted as oncogenic drivers for the development of ACKD-RCC, together with providing insight into mechanisms underlying ACD-RCC development, as well as diagnostic and treatment options.

## 1 Introduction

Acquired cystic disease-associated renal cell carcinoma (ACD-RCC) is the most common kidney tumor occurring in end-stage renal disease. It is often seen in patients who have undergone dialysis over extended periods, and is classified as an “other type of renal tumor” in the fifth edition of the WHO Classification of Renal Tumors published in 2022. To date, there are few reports on this type of tumor. Here, we report a case of ACD-RCC in which the patient had a long history of dialysis and had undergone surgery for clear cell renal cell carcinoma (ccRCC) in the opposite kidney. Interestingly, the patient showed mutations in several genes, including PTCH1, which are as yet unreported. We describe the clinical and pathological characteristics of this rare case, together with a review of the literature, aiming to enhance and expand current knowledge of ACD-RCC.

## 2 Materials and methods

### 2.1 Tissue processing, histology, immunohistochemistry, and FISH analysis

Specimens collected during surgical resection underwent fixation in 10% neutral formalin, after which they were routinely dehydrated, paraffin-embedded, sectioned (4 µm), stained with HE, and evaluated under light microscopy. The Dako Omnis automated immunohistochemical platform was used for immunohistochemical staining using the EnVision antigen retrieval method. The primary antibodies used were against AMACR (DAKO, 13H4, prediluted), CK7 (DAKO; OV-TL 12/30, prediluted), CD117 (DAKO; polyclonal, 1:500), CD10 (DAKO; 56C6, prediluted), CK20(DAKO; RCK108, prediluted), CA9 (MXB; RAB-0615; 1:100), PAX8 (MXB; EP298, prediluted), FH (Abcam; ab233394; 1:100), and SDHB (ZSGB-BIO; ZM-0162; prediluted). Probes for chromosome 3p (LBP, China) were used for fluorescence *in situ* hybridization (FISH), and fluorescence intensities were assessed using a BX51TRF fluorescence microscope (Olympus, Japan) with a single signal in 40% of cells considered to represent 3p loss.

### 2.2 Next-generation sequencing

DNA was extracted simultaneously from 10 mm-thick formalin-fixed paraffin-embedded (FFPE) sections of the tumor and adjacent normal tissues using the QIAamp DNA FFPE Tissue Kit (Qiagen, Germany). DNA concentrations were measured using Qubit assays (Qiagen, China). All procedures were conducted following the provided directions. DNA samples were utilized for pan-cancer laboratory gene testing (BGI, China), an NGS assay including 437 cancer-associated gene target sequencing panels (**Supplementary Table 1**).

## 3 Results

### 3.1 Clinical presentation

The patient was a 58-year-old man who was hospitalized due to a “right renal space-occupying lesion” after enhanced CT detection of a potentially malignant nodule in the lower pole of the right kidney. The patient underwent radical surgery for right renal cancer. He had previously received peritoneal dialysis for over nine years due to chronic renal insufficiency and uremia, and one year previously had undergone radical surgery of the left kidney due to the presence of a tumor. The postoperative pathological diagnosis of this tumor was ccRCC. The patient had had hypertension for over three years. Physical examination indicated that the patient was conscious and in good spirits, with a soft abdomen, mild tenderness in the lower abdomen, no rebound pain, no tenderness or rebound pain in the ribs, lumbar points, and ureteral points, and no edema in both lower limbs.

### 3.2 Clinicopathological features and molecular testing

**Figure 1:**
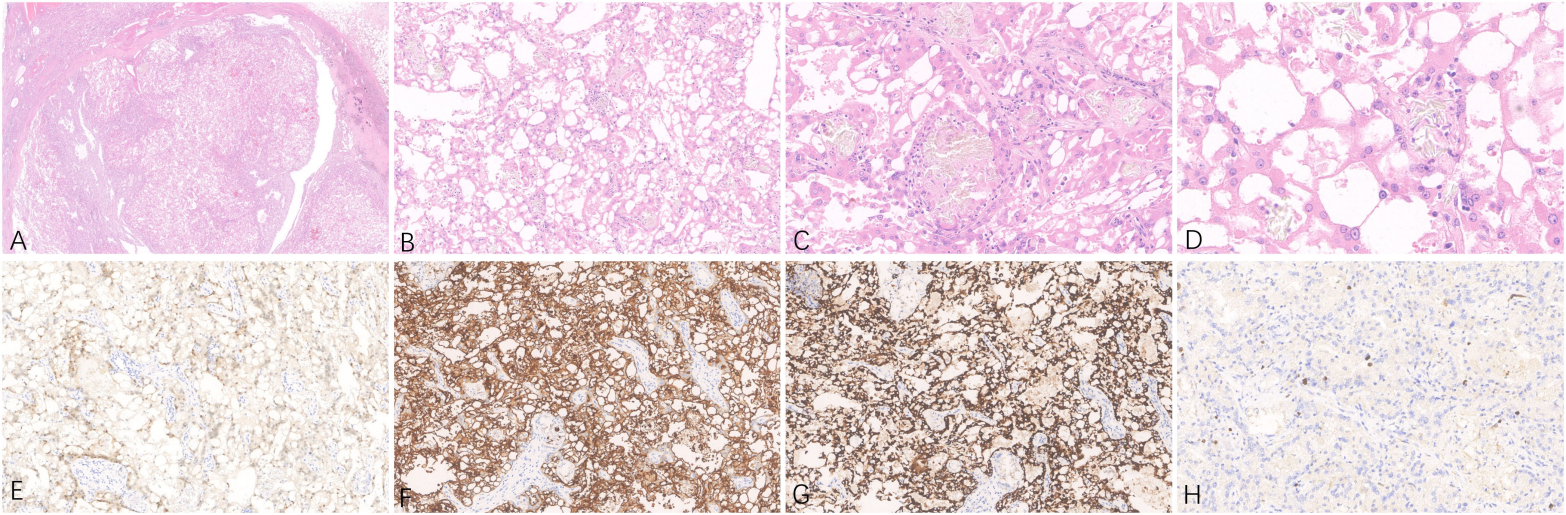
Representative hematoxylin-eosin **(A–D)** micrographs of ACD-RCC of the right kidney: **(A)** Magnification ×10; **(B)** magnification ×100; **(C)** magnification ×200; **(D)** magnification ×400. **(E)** PAX8 positivity in tumor cells, original magnification ×200. **(F)** CD10 positivity in tumor cells, original magnification ×200. **(G)** AMACR positivity in tumor cells, original magnification ×200. **(H)** Immunohistochemical Ki-67 staining showing positive cells, original magnification ×200.

The patient underwent radical surgery for right renal cell carcinoma. Postoperative gross examination revealed a kidney measuring 10.0×9.0×8.0 cm with a surrounding fat capsule that was difficult to separate. The naked kidney was 9.0×6.5×5.0 cm in size. A mass measuring 2.0×1.8×1.5 cm was found in the upper cortex of the kidney. The cut surface of the mass appeared grayish-yellow and grayish-white, with a focal cystic component. The remaining renal cortex showed multiple cystic lesions with diameters of 0.2-1.5 cm. The ureteral stump was 4.5 cm long and 0.5 cm in diameter, with smooth mucosa. Microscopic evaluation of the histology indicated clear boundaries between the tumor and surrounding normal renal tissues. The tumor cells were arranged in solid or tubular form, with abundant eosinophilic cytoplasm. The nuclei were round, and the nucleoli were obvious. Some areas showed cyst formation, with iron-hematochrome deposits in the cystic walls. Formation of local cysts of varying sizes was seen, with the tumor cells covered with eosinophilic cytoplasm. Vacuoles of varying sizes were visible in the tumor cell cytoplasm, giving the cells a cribriform or microcystic appearance. There were varying amounts of calcium oxalate crystals in both the cells and interstitial tissue. Immunohistochemistry showed that the tumor cells expressed PAX8 and CD10, while staining for CK7 and CD117 was negative, and there was no deletion of FH and SDHB proteins. The Ki-67 proliferation index was approximately 3% (**Figure 1**). The FISH results were negative for the 3p deletion probe. The NGS results identified several somatic likely pathogenic mutations, p.W844(c.2532G>A) in PTCH1, p.R2251W(c.6751C>T) in MTOR, p.P4261Q(c.12782C>A) in FAT1, p.A132E(c.395C>A) in SOS1,p.I906K(c.2717T>A) in RECQL4, and p.A207D(c.620C>A) in CDC73 (**Table 1**). Based on the histological, immunohistochemical, and NGS results, the patient was diagnosed with ACD-RCC of the right kidney.

**Table 1:** The NGS result of the mutations in ACD-RCC of the right kidney.

Mutated gene	Somatic variation	Transcript number	exon
***PTCH1***	p.W844*(c.2532G>A)	NM_000264.5	EX15
***RECQL4***	p.I906K(c.2717T>A)	NM_004260.4	EX16
***MTOR***	p.R2251W(c.6751C>T)	NM_004958.4	EX48
***CDC73***	p.A207D(c.620C>A)	NM_024529.5	EX7
***SOS1***	p.A132E(c.395C>A)	NM_005633.3	EX4
***FAT1***	p.P4261Q(c.12782C>A)	NM_005245.4	EX25

**Figure 2:**
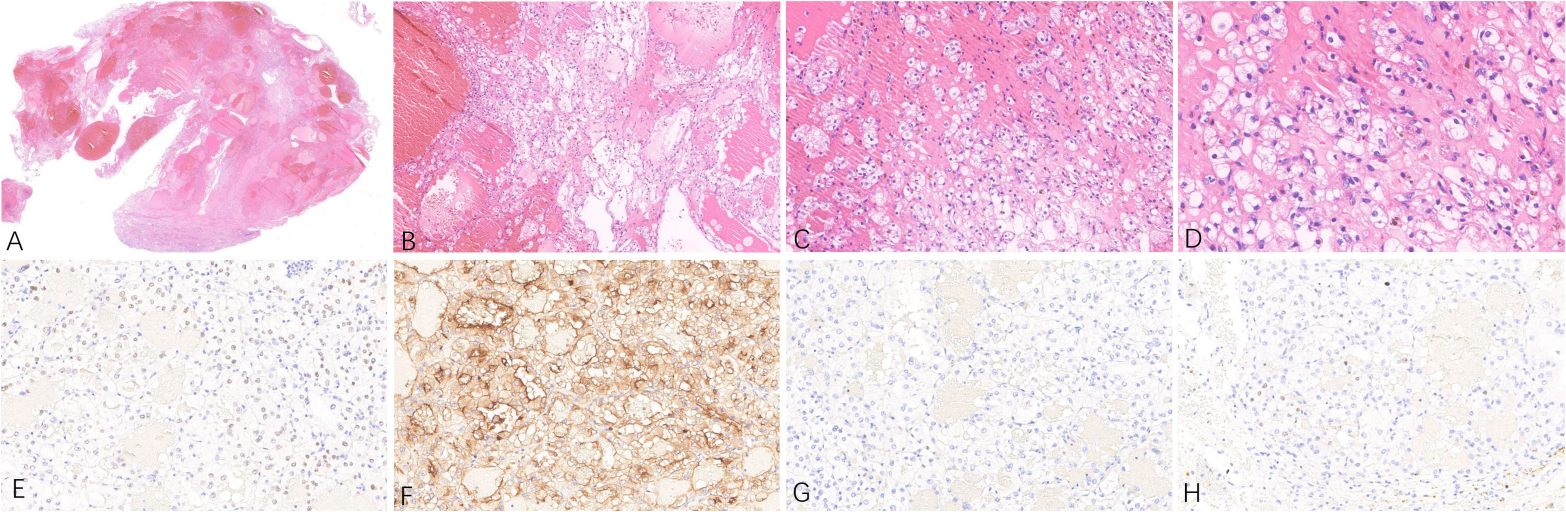
Representative hematoxylin-eosin **(A–D)** micrographs of ccRCC of the left kidney: **(A)** Magnification ×10; **(B)** magnification ×100; **(C)** magnification ×200;**(D)** magnification ×400. **(E)** PAX8 positivity in tumor cells, original magnification ×200. **(F)** CD10 positivity in tumor cells, original magnification ×200. **(G)** CK7 negativity in tumor cells, original magnification ×200. **(H)** Immunohistochemical Ki-67 staining showing positive cells, original magnification ×200.

The patient underwent radical surgery for right renal cancer one year ago. He had previously undergone radical surgery for left renal cancer as described above. The gross appearance of the left kidney indicated that the size of the kidney and the surrounding fat capsule was 8.0×6.0×5.0 cm, and the perirenal fat was not easily separated. The size of the naked kidney was 7.0×5.0×4.0 cm. A mass measuring 3.5×2.0×1.5 cm was seen in the upper cortex of the kidney. The cut surface of the tissue appeared multicolored, with a medium texture. The histological morphology showed clear demarcation between the tumor tissue and normal renal tissue. The cytoplasm of the tumor cells was clear and the interstitium contained a network of thin-walled blood vessels with prominent nucleoli. No obvious vacuoles were seen in the cytoplasm, and no calcium oxalate crystals were observed in the interstitium. The immunohistochemical results showed that tumor cells expressed PAX8, CD10, and CA9, while staining for CA9, CK7, and CD117 were negative, and the FH and SDHB proteins were not deleted. The Ki-67 proliferation index was approximately 5% (**Figure 2**). NGS detected the c444del (p.F148Lfs*11) pathogenic mutation in the VHL gene (**Table 2**). Considering the histological, immunohistochemical, and NGS results, the diagnosis of the left renal tumor was ccRCC.

**Table 2:** The NGS result of the mutations in ccRCC of the left kidney.

Mutated gene	Somatic variation	Transcript number	exon
***VHL***	c.444del(p.F148Lfs*11)	NM_000551.3	EX2
***ARID1A***	c.1501C>T(p.Q501*)	NM_139135.4	EX3
***EPHA3***	c.43G>C(p.V15L)	NM_005233.6	EX1
***MTOR***	c.5101A>G(p.M1701V)	NM_004958.4	EX36

Analysis of the mutations in the left and right renal tumors indicated that there were no significant shared mutations, and thus both were primary tumors. The final diagnosis of the tumor in the right kidney was ACD-RCC and that of the tumor in the left kidney was ccRCC.

## 4 Discussion

ACD-RCC is a malignant epithelial-derived tumor that originates from renal tubules and tends to occur in patients with acquired cystic kidney disease. Patients with acquired cystic disease have a much greater risk of developing these tumors relative to the general population (1). Most cases occur in male patients, usually after long-term dialysis. Patients are usually asymptomatic and are occasionally found during dialysis (2, 3). In the present case, the patient was male with a history of long-term dialysis and surgery for contralateral ccRCC. He showed no obvious clinical symptoms, and the suspicious lesion in the right kidney was detected on CT during follow-up. The pathological examination after surgery confirmed the diagnosis of ACD-RCC.

Histologically, ACD-RCC often presents with a diversity of tumor structures, including tubular, papillary, tubular papillary, microcystic, and solid structures. The tumor cells are characterized by eosinophilic cytoplasm, large nuclei, and prominent nucleoli. Intracellular lumens and vacuoles are common, and intracytoplasmic vacuoles cause characteristic cribriform structures in the tumor. Calcium oxalate crystals are also frequently observed in the tumor cells or interstitium in varying amounts (4, 5). Calcium oxalate crystal deposition is a unique morphological feature of ACD-RCC (6) and varying amounts are commonly seen. This calcium oxalate crystal deposition is not generally observed in other types of renal tumors, indicating the importance of this feature in the diagnosis of ACD-RCC. In some cases of ACD-RCC, tumor cells have clear cytoplasm (7), and in a few cases, sarcomatoid differentiation or rhabdoid features may be apparent (8). In terms of immunophenotype, tumor cells express PAX8, CD10, and P504S, while CK7, CD117, and GATA3 are negative (5, 9). The present rare case of ACD-RCC showed obvious eosinophilic cytoplasm in the tumor cells, together with calcium oxalate crystals in both the tumor cells and interstitium, and positive expression of PAX8, CD10, and P504S in the tumor cells. Both the histopathological and immunophenotypic features support the pathological diagnosis of ACD-RCC. In terms of differential diagnosis, the pathological diagnosis of ACD-RCC in the right kidney in the present case mainly required differentiation from ccRCC, FH-deficient renal cell carcinoma, and SDHB-deficient renal cell carcinoma. The tumor cells in this case had eosinophilic cytoplasm, vacuoles in the cytoplasm, and negative FISH detection of probes associated with 3p deletion. NGS did not detect mutations in genes such as VHL, thus excluding the pathological diagnosis of ccRCC. Immunohistochemical analysis of the tumor cells, in this case, showed no deletion of the FH or SDHB proteins, thus excluding the pathological diagnosis of FH-deficient renal cell carcinoma and SDHB-deficient renal cell carcinoma (10, 11).

In terms of genetic characteristics, ACD-RCC tumors are commonly associated with gains of chromosomes 3, 16, 7, and 17, with the most common gain being on chromosome 3. Abnormalities on chromosomes 3 and 16 may be closely associated with tumorigenesis. while deletions on chromosomes 9 and 14 may be linked with the emergence of more aggressive sarcomatoid features in tumors (8). In 2020, Shah et al. found that 4 out of 5 cases of ACD-RCC had mutations in the KMT2C gene and 3 had mutations in TSC2, as shown by second-generation sequencing. Mutations in KMT2C, a gene associated with chromatin modification, may be carcinogenic drivers in the development of ACD-RCC. and mutations in TSC2 may play a role in tumor progression of ACD-RCC in some patients with acquired cystic kidney disease (12). NGS showed that this case of ACD-RCC had mutations in the PTCH1, MTOR, FAT1, SOS1, RECQL4, and CDC73 genes and did not have TSC2 and KMT2C gene mutations. The PTCH1 gene mutation in this case belong to the variants of potential Clinical Significance. Others include MTOR, FAT1, SOS1, RECQL4, and CDC73 genes mutation in this case belong to variants of Potential Clinical Significance. These genetic mutations have not been reported previously and are complementary to the pathogenesis of ACD-RCC. Specifically, we detected mutations in PTCH1 in the tumor tissue of the reported case. The PTCH1 gene encodes the patched-1 receptor protein, and the protein encoded by the Sonic Hedgehog gene is the ligand for this receptor. Both play an important role during early development. PTCH1 functions as a tumor suppressor gene and in the absence of Sonic Hedgehog, PTCH1 can prevent cell growth and proliferation. Conversely, in the presence of Sonic Hedgehog, PTCH1 does not inhibit cell proliferation (13).

Studies have shown that methylation of the PTCH1 gene may be associated with the development of certain tumors. PTCH1 is overexpressed in many recurrent and metastatic cancers and due to its function as a multidrug transporter, can promote the efflux of chemotherapy drugs such as doxorubicin, leading to chemotherapy resistance. The drug vismodegib, which is approved by the FDA for treating basal cell carcinoma, is an inhibitor of the Hedgehog pathway (14) and binds to and inhibits the transmembrane protein Smoothened, encoded by the SMO gene, which is involved in Hedgehog signaling. Studies have found that vismodegib is well-tolerated, mainly with grade 1-2 side effects, and significant responses have been observed in patients with mutations in SMO and PTCH1 (15). The conventional treatment for ACD-RCC remains partial or complete nephrectomy, and there are currently no specific chemotherapy drugs for ACD-RCC. However, vismodegib, which targets issues associated with PTCH1 mutations, would be expected to bring hope to these patients. The abnormal activity of SHH signaling was observed in a broad range of malignancies include renal cell carcinoma. It has been reported that increased expression of SHH, SMO, Gli1 and PTCH1 genes in tumor ccRCC tissues, suggest the involvement of SHH signaling in ccRCC initiation (16). Kowalik et al. reported one case of chromophobe renal cell carcinoma with PTCH1 mutation and additionally signalize the role of PTCH1 in renal oncogenesis (17).

In terms of prognosis, most ACD-RCC cases have a good prognosis, due to the low-grade malignancy of the tumor, although there are reports of tumor recurrence or distant metastasis. The presence of sarcomatoid differentiation often indicates a poor prognosis. The patient with ACD-RCC reported in our study had a history of ccRCC tumor surgery one year before. Currently, the patient has undergone bilateral nephrectomy. The second-generation sequencing results confirmed that the bilateral renal tumors had different genetic mutations. The ACD-RCC in the right kidney had mutations in PTCH1 and MTOR, while the ccRCC in the left kidney had mutations in VHL. The patient’s left and right renal tumors were thus double primary tumors, an extremely rare occurrence that we have reported for the first time. The patient is currently being followed up with good recovery and has not experienced tumor recurrence or metastasis.

In conclusion, the present study describes a rare case of a patient with a long history of dialysis, a right renal tumor with ACD-RCC and PTCH1 gene mutations, and a surgical history of left renal ccRCC. It is extremely unusual for ACD-RCC and ccRCC to occur in a single patient. This is also the first report of mutations in PTCH1 in association with ACD-RCC and this case report thus expands the understanding of ACD-RCC.

## Data availability statement

The original contributions presented in the study are publicly available. This data can be found here: https://www.ncbi.nlm.nih.gov/sra/PRJNA1067705, accession number: PRJNA1067705.

## Ethics statement

The studies involving humans were approved by the Ethics Committee of Ruijin Hospital. The studies were conducted in accordance with the local legislation and institutional requirements. Written informed consent for participation in this study was provided by the participants’ legal guardians/next of kin. Written informed consent was obtained from the individual(s) for the publication of any potentially identifiable images or data included in this article.

## Author contributions

LZ: Funding acquisition, Writing – original draft. HX: Writing – original draft, Methodology. YL: Data curation, Writing – original draft. XL: Writing – original draft. CL: Writing – review & editing. XY: Writing – review & editing. CW: Funding acquisition, Writing – review & editing.
