# Supplementary material for: Acquired cystic disease-associated renal cell carcinoma with PTCH1 mutation: a case report
Source: Front Oncol. 2024 Feb 2;14:1349610. doi: 10.3389/fonc.2024.1349610 (PMC10870146; doi:10.3389/fonc.2024.1349610)
Supplement: Supplementary file 1 [file Table_1.docx]

| Supplementary Table 1. 437 cancer-associated genes included in NGS. | | | | | |
| --- | --- | --- | --- | --- | --- |
| ABCB1(MDR1) | ABCC2(MRP2) | ADGRB3(BAI3) | ADH1B | AFDN(MLLT4) | AIP |
| AKT1 | AKT2 | AKT3 | ALDH2 | ALK | AMER1(FAM123B) |
| APC | AR | ARAF | ARID1A | ARID1B | ARID2 |
| ARID5B | ASCL4 | ASXL1 | ATF1 | ATIC | ATM |
| ATR | ATRX | AURKA | AURKB | AXIN2 | AXL |
| B2M | BAD | BAK1 | BAP1 | BARD1 | BAX |
| BCL2 | BCL2L11(BIM) | BCR | BIRC3 | BLM | BMPR1A |
| BRAF | BRCA1 | BRCA2 | BRD4 | BRIP1 | BTG2 |
| BTK | BUB1B | CASP8 | CBL | CBLB | CCN6(WISP3) |
| CCND1 | CCNE1 | CD274(PD-L1) | CD74 | CDA | CDC73 |
| CDH1 | CDK10 | CDK12 | CDK4 | CDK6 | CDK8 |
| CDKN1A | CDKN1B | CDKN1C | CDKN2A | CDKN2B | CDKN2C |
| CEBPA | CEP57 | CHD4 | CHD8 | CHEK1 | CHEK2 |
| CREBBP | CRKL | CSF1R | CTCF | CTLA4 | CTNNB1 |
| CUL3 | CUX1 | CXCR4 | CYLD | CYP19A1 | CYP2A13 |
| CYP2A6 | CYP2A7 | CYP2B6 | CYP2C19 | CYP2C9 | CYP2D6 |
| CYP3A4 | CYP3A5 | CYSLTR2 | DAXX | DDR2 | DENND1A |
| DHFR | DICER1 | DLL3 | DNMT3A | DOT1L | DPYD |
| DTL(CDT2) | DUSP2 | EGFR | EIF1AX | EML4 | EMSY(c11orf30) |
| EP300 | EPAS1 | EPCAM | EPHA2 | EPHA3 | EPHA5 |
| ERBB2(HER2) | ERBB3 | ERBB4 | ERBIN(ERBB2IP) | ERCC1 | ERCC2 |
| ERCC3 | ERCC4 | ERCC5 | ESR1 | ETV1 | ETV4 |
| ETV5 | ETV6 | EWSR1 | EXT1 | EXT2 | EZH2 |
| EZR | FANCA | FANCC | FANCD2 | FANCE | FANCF |
| FANCG | FANCI | FANCL | FANCM | FAT1 | FBXW7 |
| FGF19 | FGFR1 | FGFR2 | FGFR3 | FGFR4 | FH |
| FLCN | FLT1(VEGFR1) | FLT3 | FLT4 | FOXA1 | FOXL2 |
| FOXO1 | FOXP1 | FRG1 | GATA1 | GATA2 | GATA3 |
| GATA4 | GATA6 | GNA11 | GNAQ | GNAS | GREB1 |
| GREM1 | GRIN2A | GRM3 | GRM8 | GSTM1 | GSTM4 |
| GSTP1 | GSTT1 | HDAC2 | HDAC9 | HGF | HLA-A |
| HNF1A | HNF1B | HOXB13 | HRAS | IDH1 | IDH2 |
| IFNA6 | IFNB1 | IFNE | IFNG | IFNGR1 | IFNGR2 |
| IGF1R | IGF2 | IKBKE | IKZF1 | IL7R | INPP4B |
| IRF2 | JAK1 | JAK2 | JAK3 | JARID2 | JUN |
| KDM5A | KDR(VEGFR2) | KEAP1 | KIF1B | KIT | KITLG |
| KLLN | KMT2A(MLL) | KMT2B | KMT2C | KMT2D(MLL2) | KRAS |
| LHCGR | LMO1 | LRP1B | LYN | LZTR1 | MAP2K1(MEK1) |
